# Attitudes of Nursing Students towards Vaccination and Other Preventive Measures for Limitation of COVID-19 Pandemic: Cross-Sectional Study in Three European Countries

**DOI:** 10.3390/healthcare9070781

**Published:** 2021-06-22

**Authors:** Nevenka Kregar Velikonja, Beata Dobrowolska, Sanja Stanisavljević, Karmen Erjavec, Vislava Globevnik Velikonja, Ivan Verdenik

**Affiliations:** 1 Faculty of Health Sciences, University of Novo Mesto, 8000 Novo Mesto, Slovenia; karmen.erjavec@uni-nm.si (K.E.); vislava.velikonja@guest.arnes.si (V.G.V.); ivan.verdenik@guest.arnes.si (I.V.); 2 Department of Nursing Management, Faculty of Health Sciences, Medical University of Lublin, 20-081 Lublin, Poland; beata.dobrowolska@umlub.pl; 3 The College of Health Sciences, Academy of Applied Studies Belgrade, 11000 Belgrade, Serbia; stanisavljevicsanja@gmail.com; 4 Division for Obstetrics and Gynaecology, University Medical Centre Ljubljana, Šlajmerjeva 4, 1000 Ljubljana, Slovenia

**Keywords:** nursing students, COVID-19, preventive behavior, vaccination intention, advising vaccination

## Abstract

Several preventive measures have been applied to limit the COVID-19 pandemic, including successful the development and introduction of vaccines. The aim of this study was to investigate adherence to preventive measures and vaccination intentions among nursing students in three European countries and the factors associated with vaccination intention and advising vaccination. A cross-sectional study using convenience/snow-ball sampling strategy was performed in Slovenia, Poland, and Serbia between 12 February and 5 March 2021. Data from 872 eligible respondents were analyzed (mean age 23.5 ± 6.5 years, 89% female). Higher adherence to preventive behavior was declared by those working in healthcare (*p* < 0.001), engaged in COVID-19 departments (*p* < 0.001), had not had the disease yet (*p* < 0.001), and had children (*p* = 0.01). Those groups also expressed higher vaccination intention and advised vaccination to others. Higher vaccination intention and advising vaccination were mostly associated with belief in benefits of vaccine, trust in institutions, perceived effectiveness of vaccine, influence of social environment, protection of patients and perceived health care professionals’ duty. Fear of side effects and general refusal of vaccines are the main reasons for vaccination hesitancy. The results of the study indicate how higher education institutions can support the development of appropriate professional attitudes and behaviors among nursing students.

## 1. Introduction

Several preventive measures are being applied during the COVID-19 pandemic to reduce SARS-CoV-2 transmission [1,2]. Vaccination against COVID-19 is expected to be the most efficient preventive measure for limiting the pandemic. Vaccines against SARS-CoV-2 became available at the end of 2020, and healthcare workers (HCWs) were in many countries among the first groups to be vaccinated [3,4]. The success of a vaccination program depends on the uptake rates among the general population and especially among HCWs, who are important for vaccination advocacy [5,6]. Apart from being at a higher risk of becoming infected with SARS-CoV-2 than the general population [7,8,9], HCWs are also potential transmitters of the virus in the clinical setting [10], where they work with the most susceptible population, i.e., the elderly, and those with certain underlying medical conditions, which require more attention and care [11,12]. Studies have shown that vaccine acceptance and intention to be vaccinated against COVID-19 are higher among HCWs than in the general population, mainly because of a higher perceived risk of infection with SARS-CoV-2 [3,13,14]. Compliance with preventive measures is influenced by individuals’ attitudes and perceived vulnerability to disease [15]. Also, in the time of the COVID-19 pandemic greater compliance with preventive behavior has been found in individuals who experience greater psychological distress, are more anxious, and express greater perceived infectability and germ aversion [16]. Vaccination acceptance and vaccination hesitancy are influenced by several factors, such as fear of adverse side effects and vaccine safety, perceived ineffectiveness of vaccine, poor information regarding illness/vaccine, perceived low risk of contracting illness, fear of needles, perceived low severity of illness, etc. [17]. 

HCWs are recognized as the most important sources of information regarding health prevention issues and the strongest determinants of people’s vaccination decisions [6,18,19,20]. Research on factors influencing HCWs vaccination acceptance and vaccination advocacy in cases of influenza, HPV, hepatitis B and other contagious diseases revealed that the knowledge about particular vaccines, their efficacy and safety, helped to build HCWs own confidence in vaccines and their willingness to recommend vaccines to others. The importance of societal endorsement and support from colleagues was also reported [17,20,21]. Therefore, it is important to know HCW’s opinions and vaccination intentions, and to understand how key sociodemographic factors are related to vaccination intentions in the context of the COVID-19 pandemic. Nursing students are highly involved in work in the health sector due to practical training and to their actual employment in the health sector. Moreover, they are educated and trained for an important role in health promotion and prevention [22].

The studies on preventive behavior of nursing students in the time of the COVID-19 pandemic have mostly revealed their high level of adherence to preventive measures [23,24,25,26]. A study in Spain revealed a lack of knowledge of basic measures to prevent the transmission of the virus at both community and hospital levels [27]. Vaccination intention studies among nursing students are scarce. In a study in the US, only 45% nursing students intended to be vaccinated against COVID-19 [28]. 

Due to the future professional roles of nursing students and their position in health care teams, this study aimed to analyze the preventive behaviour, vaccination acceptance and vaccination advocacy of nursing students in three European countries, as well as the factors influencing their vaccination intention. The survey was performed in Slovenia, Poland and Serbia, the countries belonging to Central and Eastern Europe [29] with comparable system of nursing education, organized according to the EU directive 2005/36/EC [30]. Therefore, the results of the survey may add to the literature regarding European nursing students’ attitudes regarding preventive behaviors and vaccination acceptance and advocacy, as well as indicate future research directions and practical actions that should be undertaken. We hypothesized that working experience would be positively associated with higher adherence to preventive measures, higher vaccination intention and vaccination advocacy. Responsibility towards patients was expected to be among most important motivational factors for vaccination.

## 2. Materials and Methods

### 2.1. Study Design

A cross-sectional study with a web-based survey on COVID-19 preventive measures, vaccination intention, and attitudes towards vaccination was conducted among nursing students in three Central and Eastern European countries Slovenia, Poland, and Serbia.

The online survey was prepared using web based platform 1 ka [31], and distributed among nursing students of different higher education institutions (HEI) via HEI contact lists to approximately 600 students in each country, and further distributed using the snow-ball sampling method [32,33,34]. Students were asked to share the survey link with their colleagues willing to take part in the study. Respondents were asked to complete a self-administered, structured electronic questionnaire. The time frame of the survey activity in the participating countries was 12 February–5 March 2021.

The study protocol was reviewed and approved by the Ethical Committee at the Faculty of Health Sciences, University of Novo mesto (University ethical approval No. FZV-98/2020). Students were informed about the aim of the study, anonymity of results, and of the possibility to withdraw from the study at any time. This information was provided at the beginning of a web-based survey.

### 2.2. Participants

Nursing students of the 1st and the 2nd level of education (Bachelor and Master’s degrees) were eligible to take part in the survey, both attending full or part-time studies. Respondents that did not identify themselves as nursing students and those with missing answers on key items were excluded. The questionnaire was fully completed by 872 respondents who fulfilled the inclusion criteria: 252 were from Slovenia, 353 from Poland, and 267 from Serbia (Table 1). There were 775 (89%) female and 97 (11%) male respondents. Their average age was 23.5 ± 6.5 years. As many as 265 (30.4%) respondents were already employed in the health sector, 78 (8.9%) worked occasionally, 529 (60.7%) were involved in clinical work only during clinical training. More than a quarter of respondents already had COVID-19, and a bit less were already vaccinated.

### 2.3. Research Instrument 

The questionnaire included 21 questions containing 134 variables on demographic characteristics of the participants (age, gender, level of education, work engagement, if they already had COVID-19), adherence to preventive behavior, vaccination intention, acceptance and attitudes, as well as the psychological burden, anxiety, germ aversion and perceived infectability. It took 10 to 14 min for the respondents to complete the survey. 

Parts of the instrument were as follows:

- *assessment of preventive behavior*: respondents evaluated their level of agreement with 10 statements describing different preventive measures from 1 (absolutely not) to 5 (absolutely yes) as described previously [16] with the additional statement ‘I regularly take care for aeration of premises’. For further analysis the average value of all statements was calculated for each respondent to obtain the preventive behavior score (Cronbach’s alpha = 0.75).
- *respondents’ vaccination intention, acceptance and attitudes*: a self-administered questionnaire composed of 34 statements was used to evaluate respondents’ attitudes and beliefs regarding COVID-19 (listed in Appendix A Table A1; response options revealing respondents’ level of agreement from 1 (absolutely not) to 5 (absolutely yes)) (Cronbach’s alpha = 0.87). The statements were partially adopted from the CoVaCCs study [35] and additionally formulated to verify the main parameters influencing vaccination intention of health workers as described by Yaqub et al. [17]. Statements ‘I will definitely be vaccinated’ and ‘I personally advise people to be vaccinated’ were considered as reference statements to evaluate respondents’ ‘vaccination intention’ and their engagement in ‘advising vaccination’.
- *psychological burden*: the Thermometer for Mental Health was used to assess the psychological burden, i.e., one’s own experience of physical, emotional, psychosocial burden, and the burden of everyday life during the last 7 days [36]. Respondents had to assess these on the continuous visual scale from 0 (no burden) to 10 (extremely strong burden).
- *anxiety*: The Generalized Anxiety Disorder 7-item, GAD-7 [37] was used. The GAD-7 consists of 7 questions based in part on the DSM-IV (Diagnostic and Statistical Manual of Mental Disorders–Fourth Edition) criteria for GAD, and reflects the frequency of symptoms during the preceding 2-week period; for each symptom queried it provides the following response options: “not at all”, “several days”, “over half the days” and “nearly every day”, and these are scored 0, 1, 2 or 3, respectively (Cronbach’s alpha = 0.92).
- *perceived infectability and germ aversion*: The Perceived Vulnerability to Disease Questionnaire, PVDQ, developed by Duncan et al. [15] and adopted to be more reflective of current reality [16], a 15-item self-report on a 7-point scale response (with endpoints labeled as “strongly disagree” and “strongly agree”) was used. It measures two factors: perceived infectability (assesses beliefs in one’s own susceptibility to infectious diseases, e.g., “If an illness is going around, I will get it”; 7 items) (Cronbach’s alpha = 0.68) and germ aversion (assesses emotional discomfort in contexts where disease-causing germs might be transmitted, e.g., “It really bothers me when people sneeze without covering their mouth”; 8 items) (Cronbach’s alpha = 0.56).

### 2.4. Statistical Analysis

The data obtained were coded, validated and analyzed using SPSS (IBM SPSS Statistics for Windows, Version 25.0, IBM Corp, Armonk, NY, USA). Descriptive analysis was used to calculate frequencies and proportions. To assess an association between variables, Spearman correlation was performed. *t*-test, Mann-Whitney test, ANOVA, and Kruskal-Wallis test were used to assess differences between the groups made according to different demographic characteristics.

For the Likert scales we used parametric versions of tests, while for two ordinal categorical questions (vaccination intention and advising vaccination), we chose non-parametric versions (Kruskal-Wallis for multiple groups, Mann-Whitney for two groups).

A *p*-value less than 0.05 was considered statistically significant. 

## 3. Results

### 3.1. Adherence to Preventive Measures and Its Dependence on Demographic Factors and Perception of the Situation

Respondents’ adherence to different preventive measures (preventive behavior, vaccination intention, advising vaccination) showed a significant dependence on different demographic parameters (Table 2). In terms of preventive behavior, the highest adherence to preventive practices was declared by respondents from Slovenia, those who worked in healthcare, were engaged in COVID-19 departments, did not have the disease yet, and those who had children. Compared to women, men reported a higher intention to be vaccinated and were more likely to advise others to get vaccinated. These two parameters were rated highest by Polish respondents who worked in healthcare, worked in COVID-19 departments, had not been ill yet, and had children.

Scores, revealing respondents’ perception of the situation in time of the pandemic showed an association with different demographic parameters (Table 2). Working in COVID-19 departments was not associated with different perception scores; only the anxiety score showed an association with a living arrangement: higher level of anxiety was observed in those sharing a household with older people. At a cut-off of 8 on the GAD-7 as proposed by different reviewed expert opinions [38] in order to optimize the sensitivity without compromising specificity for assessing generalized anxiety disorder, anxiety was present in 35 % of respondents (43% in Poland, 26% in Slovenia, 33% in Serbia).

**Figure 1:**
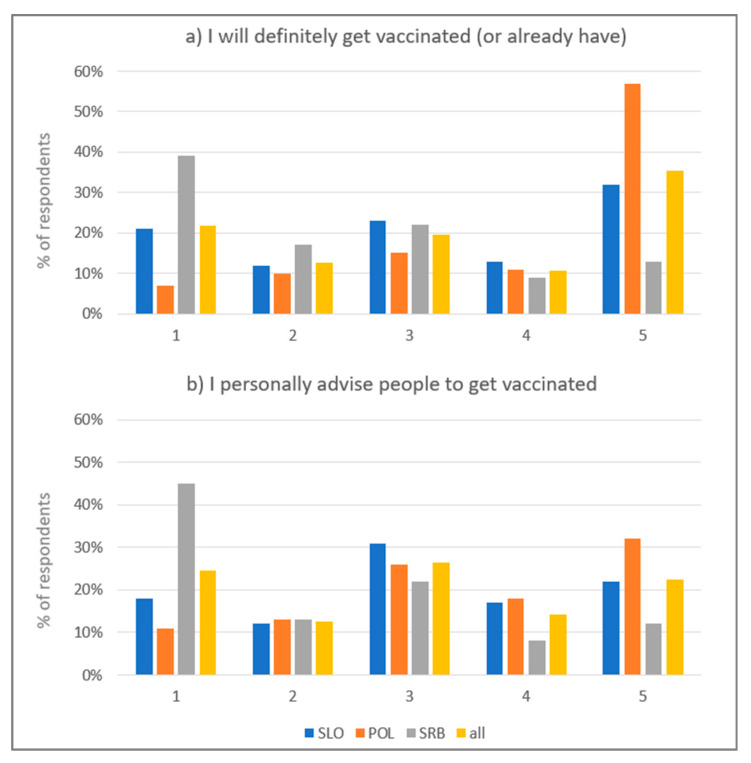
Percentage of respondents declaring different intentions regarding being vaccinated (**a**) and their engagement in advising people to be vaccinated (**b**) (1—I absolutely disagree; 5—I absolutely agree; SLO—Slovenia, POL—Poland, SRB—Serbia).

Overall, 35% of respondents declared definite intention to be vaccinated (the percentage was the highest in Poland—57%, and the lowest in Serbia—13%) and 22% definite rejection of vaccination (the percentage was the lowest in Poland—7%, and the highest in Serbia—39%) (Figure 1a). 22% of respondents declared absolute agreement with the statement about their engagement in advising vaccination (the percentage was the highest in Poland—32%, and the lowest in Serbia—12%), and 24% absolute disagreement (the percentage was the lowest in Poland—11%, and the highest in Serbia—45%) (Figure 1b).

A significant positive correlation was observed between preventive measure scores, but this correlation was strong only between vaccination intention and advising vaccination (r = 0.74). Perception scores revealed very low grades of correlation with preventive measure scores, except for germ aversion which was in moderate significant correlation with preventive behavior score (Table 3).

### 3.2. Attitudes and Beliefs Associated with Vaccination Intention and Advising Vaccination 

The most important attitudes and beliefs associated with vaccination intention and with advising vaccination were belief in benefits of the vaccine (r = 0.731 and 0.712, respectively), trust in institutions (r = 0.742 and 0.693, respectively), perceived effectiveness of the vaccine (r = 0.671 and 0.622, respectively) and influence of the social environment (r = 0.650 and 0.606, respectively). In terms of advising vaccination, HCWs’ duty was the strongest factor to advise vaccination (r = 0.744). Among the listed factors, fear of side effects (r = −0.547 and −0.475, respectively) and personal general refusal of vaccination (r = −0.520 and −0.474, respectively) were the main reasons for hesitancy. Other less important factors were time and insufficient self-perceived knowledge to advise others regarding vaccination (Table 4). All items except “fear of needles” are significant at the 0.01 level. 

## 4. Discussion

This study shows the association of various demographic factors, perceptions and attitudes with the way the nursing students consider and implement preventive measures to limit the COVID-19 pandemic, with the emphasis on vaccination. The results are of great importance considering the fact that nursing students as future nursing professionals should serve as role models taking part in health promotion and health education processes, influencing societal preventive attitudes and health behaviors.

The relatively high adherence to preventive behavior was revealed among nursing students (mean score 4.0), yet only moderate vaccination intention and engagement in advising vaccination (mean scores 3.3, and 3.0, respectively) were reported. 

The highest adherence to preventive behavior was reported by Slovene students, which could be explained by the highest percentage of part time students in the Slovene cohort. Polish students reported the lowest adherence to preventive behavior but the highest vaccination intention and engagement in advising vaccination. Vaccination intention and engagement in advising vaccination were the lowest among Serbian students. This is in line with previous research confirming different vaccination attitudes in the participating countries [19,20,39,40].

Throughout the COVID-19 pandemic, HCWs have been exposed to changing work environment, influenced by requirements for the reorganization of health sector capacities, and to the burden of risk for transmission of contagious disease to patients, requests to be vaccinated, etc. 

Perceived infectability is comparable to the European average (3.48) in our study (3.50) [15,41], but germ aversion reported by our respondents was much higher (4.50) than the expected European average (3.55). This is likely to be influenced by the current epidemic situation and the real threat of the SARS-CoV-2 infection, which has been confirmed by other studies stating that the germ aversion factor is more situationally conditioned [42]. 

Psychological burden and anxiety were relatively high among respondents; the anxiety score in particular was much higher than that reported in the initial phase of the pandemic, when 20% of respondents reported the anxiety score higher than 8, which is considered as the cut-off point [16,38]. An alarming finding is that in our study, the average anxiety score was 6.9, and that 35% of respondents had GAD7 scores over 8, far exceeding the expected prevalence of anxiety disorders in Euro/Anglo cultures where it is 10.4% (7.0–15.5%) [43], and that in the initial stage of the pandemic (20.5% in the Slovene study) [16].

The important finding is that working in healthcare as well as working in a COVID-19 department is associated with higher adherence to preventive behavior, while working in a COVID-19 department alone is not associated with increased anxiety, although this parameter is severely increased in the participating cohorts. Our respondents were highly involved in working in the clinical environment during this study. Due to the transformation of educational standards in the nursing profession over the last decades, many part time nursing students are regularly employed in healthcare and study to improve their competencies and gain a higher degree of education. Depending on the organization of clinical training in the participating countries, students in Slovenia have also been appointed to clinical training during the pandemic, while in Poland, a majority of students in master’s degree programs already work in clinical settings, while full-time undergraduate students in Serbia and Poland were not engaged in clinical departments during the COVID-19 pandemic. As the more positive attitude towards vaccination, advising vaccination and preventive behavior are associated with the engagement in clinical environment, absence from the clinical environment probably contributed to lower scores of vaccination intention and advising vaccination among students from Serbia. 

Besides engagement in clinical environment, other factors such as current epidemiologic situation and availability of vaccination for students differed among the countries at the time of the survey, and might have contributed to respondents’ attitudes. Namely, nursing students were on the priority list for vaccination in Poland, vaccination was accessible but not prioritized for nursing students in Slovenia, and in Serbia, nursing students were excluded from vaccination priorities (as shown in Table 1, 42% of Polish respondents, 22% of Slovene respondents, and only 2% of Serbian respondents were vaccinated at the time of the survey). According to Worldometer data [44], in the period of the survey, the average number of new cases and deaths was increasing especially in Serbia and in Poland, while in Slovenia, the situation was slightly improving. These circumstances might have contributed to the higher vaccination intention and advising vaccination among Polish students, and to the lowest perceived infectability and anxiety of Slovene respondents.

The results of this research show a significant association among preventive measures’ scores. However, only the correlation between vaccination intention and advising vaccination was moderately strong. There was a fairly weak positive correlation between taking preventive measures and perceived infectability and germ aversion; only germ aversion correlated moderately with the preventive behavior score. The self-assessed psychological burden was in fairly weak positive correlation with vaccination intention and advising vaccination, but the correlation between the anxiety score and all three preventive measure values was close to zero. These results differ from the findings in the general population at the initial stage of the pandemic, when a higher level of preventive behavior was found in individuals who experienced greater psychological distress, were more anxious, expressed greater perceived infectability, and experienced greater germ aversion [16]. There was even a negative correlation between the anxiety score and preventive behavior score in accordance with the recently published research performed by Wang et al. [45] who investigated the association between the psychological impact of the COVID-19 outbreak and taking precautionary measures in China, and showed that greater adherence to preventive measures was linked to lower degrees of anxiety, stress and depression.

The important measure for preventing the COVID-19 spread is vaccination. In this study, 25% of respondents were already vaccinated, and 35% expressed definite intention to be vaccinated (including those who already were vaccinated). 22% would definitely not be vaccinated, and 13% would probably not be vaccinated; therefore, the remaining 65% could be considered as ‘willing to be vaccinated’, which is less than in other recent research in Europe and the US. In a study among the general populations in 7 European countries, 73.4% [46], and in the UK 76.9% [47] of respondents were willing to be vaccinated against COVID-19. In a Canadian study, more than two-thirds of crowdsourcing participants were very likely to get a COVID-19 vaccine when it became available [48]. In Far East countries, the percentages of definite vaccination intention are higher: in Malaysia, 48.2% of respondents definitely intended to be vaccinated [49], and in Hong Kong 40% of nurses intended to be vaccinated [50]. In a current Polish study among physicians, more than 94% were willing to be vaccinated, of which 88.5% as soon as possible [3]. However, these data are not directly comparable due to differences in survey designs and timeframes of conducted surveys. Nonetheless, the studies carried out so far suggest that HCWs usually have more positive attitudes towards vaccination compared to the general population [13,51], but vaccination rates among HCWs are often low [52]. Recent research shows that nurses and assistant nurses are less prone to accept vaccination against COVID-19 than physicians [53,54]. A relatively positive attitude towards vaccination but lower intention to vaccinate was also observed in our study. Some authors suggest that this is also an issue of gender, that the nursing profession is still feminized and women are less willing to be vaccinated [54]. 

Furthermore, several other factors such as different attitudes and opinions are associated with the intention to be vaccinated against COVID-19, as has been shown in studies of vaccination acceptance for other diseases e.g., flu [55] and HPV [17]. Similarly as in the CoVaCCs study [35] we have found that the intention to be vaccinated is associated with more positive general COVID-19 vaccination beliefs and attitudes, weaker beliefs that the vaccination would cause side effects or be unsafe, greater perceived information sufficiency to make an informed decision about COVID-19 vaccination, greater perceived risk of COVID-19. 

Trust in institutions is ranked first among the tested factors according to correlation with the intention to be vaccinated. Healthcare professionals are more trustful to respondents than the government. Politicization of vaccination also gives rise to mistrust and doubt in the reality of the need for vaccination [56]. In recent years, the so-called anti-vaccine movements have developed, which is also represented by some prominent health care workers and celebrities [57].

Perceived risk of contracting the disease is ranked only 11th among the tested factors in correlation with the intention to vaccinate. The infection rate among the youth population (to which our respondents mainly belong) is relatively low. Among infected young people, the disease most often manifests itself with mild clinical signs and symptoms. This can result in unrealistic optimism among young people and in attitudes such as “it can’t happen to me”, “if it happens, I will have an easy clinical picture of the disease” [58,59,60]. 

Respondents perceived their knowledge to decide about vaccination higher than the knowledge to advise others. Lack of knowledge has been shown to be associated with hesitant attitudes toward vaccination [61]. Therefore, there is urgent need for education of future health care professionals on vaccination against COVID-19. Considering the fact that they will become professionals responsible for education of the society regarding different preventive measures, including vaccination, they should possess sufficient knowledge regarding biochemical characteristics, differences and modes of action of available vaccines against COVID-19. This is especially important in times of information chaos, which is even more visible in times of a pandemic [62].

However, HCWs are also important in helping the lay population understand the preventive measures and accept vaccination. Their opinions are valued in the lay population, and so therefore they have a considerable impact on health promotion and public opinion in the context of the pandemic. Yaqub et al. reviewed the reasons for support vs. the reasons for hesitancy in advising vaccination to patients for HPV vaccination [17]. Our study revealed that similarly the attitudes such as a perceived high risk of contracting the disease, perceived severity of the disease, concerns, belief in benefits of vaccines, trust in institutions, influence of the social environment, etc. (listed in Table 4) are associated with their own vaccination intention and with advising vaccination to others. The results show that protection of patients and perception that it is HCWs’ duty to advise patients for vaccination are among the factors with the highest correlation coefficient with vaccination intention and advising vaccination. It has previously been shown that triggering altruistic motives prove to be most efficient in increasing the willingness to vaccinate. The strategy that by being vaccinated HCWs will participate in reducing the risk for individuals who cannot be vaccinated has been highlighted as the most effective by far [63]. 

### Study Limitations and Strengths

As a cross-sectional study performed in three different countries, the present study has important strengths but also some limitations. (a) The main limitation of the study was that student cohorts in different countries differed significantly according to demographic parameters such as age, gender and percentage of part time students, potentially resulting from the convenience sampling method, although this is also a general characteristic of the nursing student cohort [64]. (b) Further, at the time of the study, vaccination strategies were different among countries and were changing as new vaccines appeared on the market. Generally, students working in the health sector were high on priority lists to be vaccinated, whereas vaccination was less available to others. We therefore grouped students that were already vaccinated or definitely intend to be vaccinated in one category, although vaccine availability could influence their perceptions and attitudes. (c) It has to be considered that the survey results relay mainly on respondents’ subjective perception and opinions regarding different parameters. Although translations were validated, the nuances in meanings of translations in different languages might have resulted in different average grades of respondents’ agreement or estimation. All this must be considered in the interpretation of the comparisons of study results among the countries. (d) To keep the results simpler and to better communicate the message to the reader, we chose the use of univariate statistics. Using multivariate methods some conclusions could be stronger. (e) Cronbach’s alpha is less reliable for perceived infectability and germ aversion scores. The authors of the questionnaire [15], however, showed an acceptable level of internal consistency: for the seven items on the Perceived Infectability factor, Cronbach’s alpha = 0.87 and for the eight items on the Germ Aversion factor, Cronbach’s alpha = 0.74. This might be situation dependent as similar alphas were obtained in research in March 2020 [16]. (f) The social desirability bias should be considered as students with higher motivation in taking preventive measures might also have been more motivated to participate in the survey. (g) Lastly, in each country, students from at least three higher-education institutions (HEI) were invited to participate in the survey, but we could not estimate the dispersion of respondents among different HEI. 

Yet, the study performed in three different European countries allows for a better understanding of different factors associated with students’ preventive behavior, their willingness to be vaccinated, and their engagement in advising other people to be vaccinated. The important feature of this study is its time frame, when vaccination against COVID-19 across Europe was in the initial phase and understanding the reasons to support and to hesitate were very important to develop appropriate motivational strategies to enhance vaccination. It is also important to understand how nursing students adhere to different preventive measures one year after the pandemic.

## 5. Conclusions

This study highlights which aspects the HEI should support in the development of appropriate professional attitudes and behaviors in nursing students in terms of preventive practices, especially vaccination, and thus participate in the prevention of the SARS-CoV-2 spread. It is crucial to understand the adherence to preventive measures and vaccination intention of nursing students, as well as the factors associated with vaccination hesitancy so that their professional attitudes and behavior, including vaccination advocacy, may be improved through the educational process. With the implementation of relevant content into educational curricula, HEIs could increase the trust in vaccination and help to clarify safety concerns. It is most important that nursing students as future health professionals accept their position as role models in providing public health, especially in times of fighting new contagious diseases. 

## Figures and Tables

**Table 1:** Response rate and demographic data of included respondents.

	Slovenia	Poland	Serbia	All
Accessed the survey	369	1110	457	1936
Eligible responses	252	353	267	872
Mean age (years (SD))	27.8 (8.3)	22.7 (4.7)	20.6 (4.2)	23.5 (6.5)
*N* (%) female	194 (77%)	330 (93%)	251 (94%)	775 (89%)
*N* (%) working in healthcare	145 (58%)	86 (24%)	34 (13%)	265 (30%)
*N* (%) already had COVID-19	101 (41%)	72 (21%)	51 (19%)	224 (26%)
*N* (%) already vaccinated against COVID-19	58 (23%)	148 (42%)	7 (2%)	213 (24%)

**Table 2:** Descriptive statistics for variables defining preventive behavior (preventive behavior score, vaccination intention, advising vaccination—shaded fields) and analyzed scores reflecting respondent’s perception (germ aversion, perceived infectability, anxiety, and psychological burden) and differences in the analyzed scores according to demographic characteristics.

		*N* (%)	Preventive Measures			Perception of the Situation			
			Preventive Behavior	Vaccination Intention	Advising Vaccination	Germ Aversion	Perceived Infectability	Anxiety	Psychological Burden
Score descriptive statistics	Score range		1–5	1–5	1–5	1–7	1–7	0–21	0–10
	Score mean (SD)		4.0 (0.61)	3.3 (1.57)	3.0 (1.46)	4.5 (1.03)	3.5 (1.00)	6.9 (5.8)	4.9 (2.69)
Gender	Male	97 (11.1%)	4.1 (0.62)	3.6 (1.49)	3.3 (1.41)	4.5 (0.96)	3.3 (1.05)	5.2 (5.4)	4.2 (2.77)
	Female	775 (88.9%)	4.0 (0.61)	3.2 (1.58)	2.9 (1.47)	4.6 (1.04)	3.5 (0.99)	7.1 (5.8)	5.0 (2.66)
*t*-test	*p*		0.443	0.031 *	0.020 *	0.459	0.101	0.002	0.007
Country	Slovenia	252 (28.9%)	4.2 (0.61)	3.3 (1.52)	3.1 (1.37)	4.7 (1.02)	3.3 (0.91)	5.7 (5.7)	4.4 (2.64)
	Poland	353 (40.5%)	3.9 (0.61)	4.0 (1.33)	3.5 (1.35)	4.4 (1.04)	3.7 (1.06)	8.4 (5.9)	5.7 (2.54)
	Serbia	267 (30.6%)	4.0 (0.59)	2.4 (1.42)	2.3 (1.41)	4.6 (1.01)	3.4 (0.96)	6.2 (5.3)	4.3 (2.63)
ANOVA	*p*		<0.001	<0.001 ^#^	<0.001 ^#^	0.002	<0.001	<0.001	<0.001
Working in healthcare	Yes	265 (30.4%)	4.2 (0.55)	3.5 (1.53)	3.2 (1.42)	4.7 (1.05)	3.5 (1.04)	5.9 (5.1)	4.8 (2.52)
	Occasionally	78 (8.9%)	4.0 (0.50)	3.1 (1.58)	2.8 (1.45)	4.6 (0.89)	3.2 (0.98)	6.9 (6.2)	4.3 (2.63)
	No	529 (60.7%)	3.9 (0.64)	3.2 (1.58)	2.9 (1.47)	4.5 (1.04)	3.6 (0.98)	7.4 (6.0)	5.0 (2.76)
ANOVA	*p*		<0.001	0.015 ^#^	0.004 ^#^	0.110	0.023	0.005	0.068
Working in COVID-19 dept	regularly	118 (13.5%)	4.2 (0.58)	3.4 (1.55)	3.3 (1.38)	4.7 (1.02)	3.5 (1.02)	6.4 (5.6)	4.9 (2.50)
	occasionally	197 (22.6%)	4.1 (0.57)	3.5 (1.51)	3.1 (1.47)	4.6 (1.03)	3.5 (1.02)	6.8 (5.7)	4.9 (2.59)
	no	557 (63.9%)	3.9 (0.63)	3.1 (1.59)	2.8 (1.47)	4.5 (1.03)	3.5 (0.99)	7.0 (5.8)	4.9 (2.76)
ANOVA	*p*		<0.001	0.031 ^#^	0.013 ^#^	0.193	0.885	0.560	0.946
Had COVID-19	yes	224 (25.7%)	4.0 (0.60)	3.1 (1.43)	2.9 (1.37)	4.6 (1.02)	3.7 (0.97)	6.3 (5.5)	4.7 (2.71)
	not sure	175 (20.1%)	3.8 (0.63)	3.0 (1.61)	2.7 (1.48)	4.3 (1.02)	3.6 (1.00)	8.3 (6.1)	5.4 (2.74)
	no	466 (53.4%)	4.1 (0.60)	3.4 (1.61)	3.1 (1.48)	4.7 (1.02)	3.4 (1.00)	6.7 (5.7)	4.7 (2.63)
ANOVA	*p*		<0.001	0.024 ^#^	0.001 ^#^	<0.001	0.001	0.002	0.007
Living arrangement	alone	175 (20.1%)	4.0 (0.58)	3.6 (1.54)	3.1 (1.42)	4.5 (1.05)	3.5 (1.01)	7.2 (5.9)	5.0 (2.79)
	with older > 65	85 (9.7%)	4.0 (0.66)	3.5 (1.57)	3.1 (1.50)	4.6 (1.20)	3.8 (0.88)	8.2 (6.2)	5.4 (2.66)
	with children	96 (11.0%)	4.1 (0.59)	3.7 (1.46)	3.6 (1.35)	4.7 (1.08)	3.3 (0.99)	5.3 (5.5)	4.7 (2.57)
	with older and children	75 (8.6%)	4.1 (0.60)	3.1 (1.60)	2.9 (1.36)	4.5 (1.03)	3.5 (0.99)	6.4 (6.0)	4.5 (2.80)
	with partner or other	441 (50.6%)	3.9 (0.62)	3.0 (1.56)	2.8 (1.48)	4.6 (0.98)	3.5 (1.01)	7.0 (5.6)	4.8 (2.65)
ANOVA	*p*		0.010	<0.001 ^#^	<0.001 ^#^	0.537	0.097	0.018	0.241

* non-parametric Mann-Whitney test; ^#^ non-parametric Kruskal-Wallis test.

**Table 3:** Correlation among preventive measure scores (shaded fields) and between preventive measure scores and perception scores (perceived infectability, germ aversion, psychological burden, and anxiety).

	Spearman Correlation of Preventive Behaviour Score with: r (*p*)	Spearman Correlation of Vaccination Intention with: r (*p*)	Spearman Correlation of Advising Vaccination with: r (*p*)
Vaccination intention	0.185 (<0.001)	-	/
Advising vaccination	0.242 (<0.001)	0.740 (<0.001)	-
Germ aversion	0.468 (<0.001)	0.091 (0.010)	0.119 (<0.001)
Perceived infectability	0.130 (<0.001)	0.154 (<0.001)	0.135 (<0.001)
Anxiety	−0.079 (0.021)	0.088 (0.013)	0.062 (0.081)
Psychological burden	−0.040 (0.244)	0.163 (<0.001)	0.103 (<0.001)

**Table 4:** Attitudes and beliefs associated with vaccination intention and advising vaccination (Spearman correlation coefficient)—sorted by the strength of an association.

	I Will Definitely Be Vaccinated (or Already Have) (r)	Rank	I Personally Advise People to Be Vaccinated (r)	Rank
Trust in institutions	0.742	1	0.693	3
Belief in benefits of vaccine	0.731	2	0.712	2
Protection of patients	0.678	3	0.601	6
Perceived effectiveness of vaccine	0.671	4	0.622	4
HCWs’ duty	0.655	5	0.744	1
Influence of social environment	0.65	6	0.606	5
Responsibility	0.592	7	0.58	7
Concern	0.575	8	0.556	9
Fear of side effects	−0.547	9	−0.475	11
To avoid absenteeism/normal life	0.541	10	0.493	10
Perceived high risk if contracting illness	0.526	11	0.434	14
Vaccination refusal	−0.52	12	−0.474	12
Accessibility	0.462	13	0.451	13
Perceived severity of illness	0.415	14	0.424	15
Patient’s trust	0.388	15	0.562	8
Sufficient knowledge	0.296	16	0.322	16
Time	−0.178	17	−0.185	17
Knowledge to advise	−0.103	18	−0.101	18
Fear of needles	0.036	19	−0.019	19

## Data Availability

The data presented in this study are available on request from the corresponding author.

## Appendix A

**Table A1:** Statements for evaluation of different factors potentially associated with vaccination intention and advising vaccination.

Factors Potentially Associated with Vaccination Intention and Advising Vaccination	Statement in the Survey	Mean Value (SD) **
Perceived high risk if contracting illness	Without a coronavirus vaccination, I am likely to catch coronavirus *	2.6 (1.39)
	I believe that coronavirus would be a mild illness for me *^,#^	3.1 (1.24)
Perceived severity of illness	I think that COVID-19 is serious disease	3.9 (1.19)
	Too much fuss is being made about the risk of coronavirus *^,#^	3.1 (1.38)
Concern	I am worried about catching coronavirus *	2.6 (1.28)
	If I don’t get a coronavirus vaccination and end up getting coronavirus, I would regret not getting the vaccination *	2.9 (1.56)
Belief in benefits of vaccine	Vaccination is the most effective way to limit the COVID-19 epidemic	3.2 (1.42)
	In general, vaccination is a good thing *	3.9 (1.27)
	A coronavirus vaccine will allow us to get back to ‘normal’ *	3.2 (1.31)
To protect patients	I would get vaccinated to reduce chance that I spread the virus to other people, especially patients	3.6 (1.45)
To avoid absenteeism/normal life	I would get vaccinated so that I can normally go to work	3.0 (1.51)
	If I were vaccinated, I think I would not need to follow social distancing and other restrictions for coronavirus *	2.2 (1.21)
Accessibility	It would be very easy for me to have a coronavirus vaccination *	3.2 (1.47)
	I don’t have time to organise vaccination for me and get vaccinated	1.7 (1.10)
Fear of side effects	I would be worried about experiencing side effects from a coronavirus vaccination *	3.6 (1.35)
	A coronavirus vaccination will be too new for me to be confident about getting vaccinated *	3.1 (1.49)
Fear of needles	I am afraid of needles *	1.5 (1.08)
Sufficient knowledge	I know enough about the coronavirus illness to make an informed decision about whether or not to get vaccinated *	3.7 (1.19)
	I know enough about the coronavirus vaccine to make an informed decision about whether or not to get vaccinated *	3.3 (1.32)
Perceived effectiveness of vaccine	The efficiency of vaccines is not sufficiently proved ^#^	3.6 (1.30)
	If I get a coronavirus vaccination, I will be protected against coronavirus *	2.8 (1.31)
Trust in institutions	If a coronavirus vaccination were recommended by the Government, I would get vaccinated *	2.7 (1.44)
	If a coronavirus vaccination were recommended by a health care professional, I would get vaccinated *	3.4 (1.41)
	Widespread coronavirus vaccination is just a way to make money for vaccine manufacturers *^,#^	2.7 (1.29)
Responsibility	We are all responsible for reducing the spread of coronavirus *	4.4 (0.95)
	I think it is not responsible if health workers do not get vaccinated	3.1 (1.42)
Influence of social environment	Most people I know will get a coronavirus vaccination	2.8 (1.25)
	Most people I know don’t trust in safety of coronavirus vaccines ^#^	3.3 (1.22)
	My family would approve of my having a coronavirus vaccination *	3.5 (1.40)
HCWs duty	It is health workers’ duty to promote vaccination	3.2 (1.41)
Patients’ trust	I think that people trust my advice regarding vaccination	3.0 (1.20)
Time	I have no time to discuss and consult about vaccination	2.7 (1.29)
Knowledge to advise	I think I don’t have enough knowledge to advice others about vaccination ^#^	3.2 (1.25)
Vaccination refusal	I am categorically against use of vaccines	2.0 (1.28)

HCW = healthcare workers; * statements taken from CoVaCCs study (Sherman et al., 2020); ** Mean value was calculated from responses with response options from 1 (absolutely not) to 5 (absolutely yes), revealing respondent’s level of agreement; ^#^ reverse coding.
